# Supplementary material for: Determining the stability of genome-wide factors in BMI between ages 40 to 69 years
Source: PLoS Genet. 2022 Aug 11;18(8):e1010303. doi: 10.1371/journal.pgen.1010303 (PMC9398001; doi:10.1371/journal.pgen.1010303)
Supplement: S1 Table — (DOCX) [file pgen.1010303.s001.docx]

| Supplementary Table 1. Number of men & women with complete BMI & GWAS summary statistics at each age interval, as well as genomic inflation (λ) & SNP-based heritability (h^2^) for the GWAS analyses comprising 20 versus 40 principal components (PCs). | | | | | | | | | |
| --- | --- | --- | --- | --- | --- | --- | --- | --- | --- |
|  | Sample size |  | 20 PCs | | |  | 40 PCs | | |
| Women |  |  | λ | h^2^ | SE |  | λ | h^2^ | SE |
| 1. BMI GWAS 40-44 yrs | 18,110 |  | 1.08 | 0.26 | 0.03 |  | 1.08 | 0.26 | 0.03 |
| 2. BMI GWAS 45-49 yrs | 24,975 |  | 1.12 | 0.28 | 0.03 |  | 1.12 | 0.27 | 0.03 |
| 3. BMI GWAS 50-54 yrs | 30,149 |  | 1.12 | 0.27 | 0.02 |  | 1.12 | 0.26 | 0.02 |
| 4. BMI GWAS 55-59 yrs | 36,010 |  | 1.18 | 0.30 | 0.02 |  | 1.17 | 0.30 | 0.02 |
| 5. BMI GWAS 60-64 yrs | 48,369 |  | 1.21 | 0.25 | 0.01 |  | 1.21 | 0.25 | 0.01 |
| 6. BMI GWAS 65-73 yrs | 35,460 |  | 1.16 | 0.23 | 0.02 |  | 1.15 | 0.23 | 0.02 |
| Men |  |  |  |  |  |  |  |  |  |
| 1. BMI GWAS 40-44 yrs | 15,891 |  | 1.08 | 0.24 | 0.03 |  | 1.08 | 0.29 | 0.03 |
| 2. BMI GWAS 45-49 yrs | 20,319 |  | 1.09 | 0.28 | 0.03 |  | 1.08 | 0.17 | 0.03 |
| 3. BMI GWAS 50-54 yrs | 23,453 |  | 1.12 | 0.31 | 0.03 |  | 1.09 | 0.24 | 0.02 |
| 4. BMI GWAS 55-59 yrs | 28,881 |  | 1.16 | 0.32 | 0.02 |  | 1.12 | 0.25 | 0.02 |
| 5. BMI GWAS 60-64 yrs | 41,455 |  | 1.19 | 0.27 | 0.02 |  | 1.16 | 0.22 | 0.01 |
| 6. BMI GWAS 65-73 yrs | 35,718 |  | 1.16 | 0.24 | 0.02 |  | 1.18 | 0.31 | 0.02 |
