# Supplementary material for: Determining the stability of genome-wide factors in BMI between ages 40 to 69 years
Source: PLoS Genet. 2022 Aug 11;18(8):e1010303. doi: 10.1371/journal.pgen.1010303 (PMC9398001; doi:10.1371/journal.pgen.1010303)
Supplement: Attachment — Submitted filename: Response_to_reviewers.docx [file pgen.1010303.s002.docx]

**REVIEWER 1**

1. (There are) essential papers that ... should be discussed or tied to the current effort.

RESPONSE: We thank the reviewer for the references provided. The reviewer hoped that we may discuss them. We have included a number of the suggested reports in our revision to help answer the question of whether or not our results could be generalized to younger ages. Please see the last paragraph of the Discussion before the Limitations.

An outstanding question is whether or not our results generalize to earlier stages in life. Here, a number of reports, relying on different methods, suggest that genetic risks spanning childhood, adolescence and early adulthood likely comprise a combination of age-specific and age-invariant influences. For example, several studies have shown that the PRS for childhood BMI predicts adult BMI, metabolic outcomes and other complex traits ^80-84^. Other studies relying on twin data have reported genetic correlations between BMI assessed at shorter age intervals spanning infancy, adolescence and teenage years that are much higher compared to genetic correlations based on wider age intervals ^8,14,85,86^. This pattern is consistent with autoregressive features. Cornes et al.’s ^9^ application of autoregressive modelling found evidence of largely age-invariant genetic influences on BMI at 12, 14 and 16. The authors also reported smaller but significant age-specific genetic influences on BMI, depending on sex, at 14 and 16 years that were uncorrelated with BMI at age 12 ^9^. The pattern of age-invariant influences is consistent with the study by Couto Alves et al. ^87^, which examined BMI spanning ages 2 to 18 years and reported a robust overlap between the genetics of child and adult BMI. The same study also identified a completely distinct genetic architecture in infancy ^87^. The reports by Warrington et al. ^88^ and Felix et al. ^89^ have shown how numerous replicated adult BMI loci also reach genome-wide significance in childhood GWAS studies of BMI. In terms of other LDSR studies, Trzaskowski et al. ^20^ reported a genetic correlation between BMI at 11 and 65 years of 0.86. The same study also found that the adult PRS for BMI explained at most 10% of the phenotypic variance in childhood BMI. Combined, the findings based on twin and molecular studies suggest that variability in heritability estimates spanning childhood, adolescence and early adulthood is likely explained by combination of mostly age-invariant plus age-specific genetic influences, which could potentially be better captured by autoregressive modeling.

**2. You conclude that: "differences in BMI across age could not be explained by the accumulation of age-specific genetic influences or autoregressive processes" This is true but it would be good to reflect on what, if anything, the genetic model selected based on parsimony would mean for the phenotypic model (if anything). I for one suspect most people would conceptualize causal auto-regressive effects of BMI at the phenotypic level, would your defining challenge such a model under what conditions would it/wouldn't it?**

In the final paragraph of the Limitations, we emphasize that because this form of genomic modelling relies entirely upon aggregated GWAS summary statistics, GenomicSEM necessarily precludes the modeling of environmental influences, including environmental influences over time, and allowances for such phenomenon as genetic control of sensitivity to the environment. Were environmental risks to be modelled, then autoregression may well detect an accumulation of environmental risks over time. It is also plausible that our best fitting GenomicSEM model may not replicate if innovative approaches capable of simultaneously modeling individual genetic and environmental risks were applied.

**3. There is a further possible source of corroboration you could pursuit, your model implies a certain rg between BMI and change in weight, UKB includes a Q on recent weight change: https://biobank.ndph.ox.ac.uk/showcase/field.cgi?id=2306 and for those pprepeatedly measured you can compute actual weight change.**

This comment illustrates another limitation of the GenomicSEM methods. In the 4^th^ Limitation, we note emphasize that GenomicSEM cannot model observed phenotypic information. Consequently, without any mean information we cannot determine the fit or likelihood or latent growth or mixture distributions. Latent growth processes are theoretically very compelling and would ideal for testing strong hypotheses about the nature of mean and variance changes in BMI over time.

**4. The final GWAS I listed previously among the literature suggestions has sumstats available, we have been running simple models with those, without a clear goal in mind other than to develop some instructional examples, but you could consider integrating them into this paper into a single model (then age 6 weeks to age 70...), might be beyond the scope of this manuscript. This is actually specifically a point where GenomicSEM would complement full data SEM/GenomicSEM, genomicSEM can go beyond a single cohort, raw data SEM can allow for additional types of models.**

The sample employed by Helgeland et al. would be ideal for modeling the nature of variance and covariance over time. Obtaining access to these data was beyond the scope of our report. However, in this revision we have referenced the Helgeland et al. report in the final paragraph before the Limitations. Here again, we have reminded readers the limitations of GenomicSEM, and the need for 'full data / GenomicSEM' methods, which would also allow us to model the nature of mean and variance changes over time. We also note that an advantage of our study is that it the reliance on data from a single cohort, UKB. This minimize difference due to ascertainment, population, etc..

**6. The discussion could use a clearer discussion of the limitation of pseudo longitudonal modeling in general, when do we expect it to fail etc.**

We have now emphasized (some of) the conditions where our use of pseudo longitudinal modeling is likely to fail in the Limitations. Importantly, we have argued that pseudo longitudinal modeling is likely to fail under the same circumstances as standard cross-sectional GenomicSEM analyses since both approaches depend critically upon the GWAS summary statistics being derived from a homogenous ethnic group. In the first limitation we now say,

… the BMI data used here were not repeated measures, but pseudo longitudinal. This approach assumes no year of birth or cohort-related genetic heterogeneity. Until now, GenomicSEM reports have typically leveraged LDSR-derived genetic covariances in the context of cross-sectional hypotheses. Our pseudo longitudinal modeling is not unlike standard cross-sectional GenomicSEM analyses. Both approaches depend upon the GWAS summary statistics being derived from a homogenous ancestral group. There is also no requirement for summary statistics to be based on the same subjects. It remains important to reduce the likelihood that our age-specific GWAS results comprised subjects from heterogenous populations. This is important because cohorts can have different BMI heritability, different environmental influences on BMI, or differences in the genetic control of sensitivity to the environment, which can bias the covariance estimates. Danish and Swedish twin studies have illustrated differential heritability by showing how increases in mean BMI in successively younger cohorts has been accompanied by increasing genetic variance ^90,91^. Therefore, to determine if cohort effects existed, we inspected the LDSR genetic correlations between the youngest and oldest age tranches i.e., two maximally age-discrepant samples of unrelated individuals. Here, the r_g_ was 0.97 (see Table 1), which suggests that the likelihood of any cohort-related genetic heterogeneity was minimal. Note, we also performed GWAS on a subset of men (N=8,337) and women (N=7,681) with repeated BMI measures at any time from 48 to 61 years and then from 62 to 72 years. Here, the test-rest LDSR correlations were r_g_=0.99 (p=1.04e-03) and r_g_=0.95 (p=1.68e-04) for men and women respectively. Thus, genetic correlations were very high between *and* within subjects.In another attempt to reduce the possibility of systematic differences between putative subpopulations in terms of allele frequencies, we re-ran the GWAS analyses using 40 PCs as covariates. As shown in Supplementary Table 1, there were only very minor differences in genomic inflation and SNP heritability between the 20 versus 40 PC results. Therefore, not only did genomic inflation remain the same regardless of the number of PCs, it did not change across age intervals. These results further reduce the likelihood of birth or cohort-related genetic heterogeneity.

REVIEWER 2

**1. The age range is limited -- only adults. It is not clear that the results is surprising given that. Previous results (Winkler et al) found lower genetic correlation between age groups of less and more than 50. It would be useful to look at younger individuals.**

We agree with this comment. Therefore, in the revised Limitations, we make clear that our results may not necessarily generalize to GWAS samples spanning different age intervals, especially samples assessed during infancy. Instead, our results apply to samples starting early mid-life. Our revision explicitly mentions the generalizability of our results. Please see the 3^rd^ paragraph in the Discussion,

… a number of reports, relying on different methods, suggest that genetic risks spanning childhood, adolescence and early adulthood likely comprise a combination of age-specific and age-invariant influences. For example, several studies have shown that the PRS for childhood BMI predicts adult BMI, metabolic outcomes and other complex traits ^80-84^. Other studies relying on twin data have reported genetic correlations between BMI assessed at shorter age intervals spanning infancy, adolescence and teenage years that are much higher compared to genetic correlations based on wider age intervals ^8,14,85,86^. This pattern is consistent with autoregressive features. Cornes et al.’s ^9^ application of autoregressive modelling found evidence of largely age-invariant genetic influences on BMI at 12, 14 and 16. The authors also reported smaller but significant age-specific genetic influences on BMI, depending on sex, at 14 and 16 years that were uncorrelated with BMI at age 12 ^9^. The pattern of age-invariant influences is consistent with the study by Couto Alves et al. ^87^, which examined BMI spanning ages 2 to 18 years and reported a robust overlap between the genetics of child and adult BMI. The same study also identified a completely distinct genetic architecture in infancy ^87^. The reports by Warrington et al. ^88^ and Felix et al. ^89^ have shown how numerous replicated adult BMI loci also reach genome-wide significance in childhood GWAS studies of BMI. In terms of other LDSR studies, Trzaskowski et al. ^20^ reported a genetic correlation between BMI at 11 and 65 years of 0.86. The same study also found that the adult PRS for BMI explained at most 10% of the phenotypic variance in childhood BMI. Combined, the findings based on twin and molecular studies suggest that variability in heritability estimates spanning childhood, adolescence and early adulthood is likely explained by combination of mostly age-invariant plus age-specific genetic influences, which could potentially be better captured by autoregressive modeling.

**2. Many researchers are not familiar with that and the specialized nomenclature used make it difficult to assess the methodology. It would be very useful-- and required -- to spell out the statistical model to explain things, e.g., what "innovations" are.**

**&**

In the Methods, under Genomic structural equation modelling, we have elaborated on the definition of innovations,

…in Figure 1, innovations at each assessment reflect novel, time- or age-specific genetic or environmental influences, which are uncorrelated with previous genetic influences.

At the end of the same paragraph, we also point out that,

Technically, this first innovation includes all genetic variance accumulated up to this first age interval. Finally, we fitted a factor analysis comprising a single factor

This elaboration also appears in the revised caption for Figure 1 and in Table 1 notes.

**3. Also, it is not clear how the GWAS were used. Only for computing genetic correlations and heritability? (maybe it was clear, I just want to make sure). Then the Lavann software used these to construct and over all covariance matrix?**

The Introduction now includes a paragraph providing a better context and clearer description of the methods used. This will be helpful for readers who are unfamiliar with molecular based heritability, genetic correlations, and structural equation modeling. The Methods also includes some minor edits to make the whole approach clearer.

**4. I was expecting to see some results estimating genetic associations of specific variants known to be highly associated with BMI, meaning, to look at their effects over time.**

The nature of this approach is its reliance on genome-wide estimates of heritability and genome-wide covariation between phenotypes. Thus, this approach provides a bird's-eye view of the overall genetic correlations between BMI measured over time. It is not designed to determine if a particular locus contributes to more versus less variance at a time interval or intervals. Here, we note that effect sizes for most genome-wide significant loci are very small. For example, the SNP with the largest effect residing in the FTO gene accounts for ~0.4% of the total variance in BMI. While the samples sizes in the current study are large and well powered for examining aggregate effects within an age tranche, there is insufficient power to test age-specific effects for specific loci, including FTO. Also, testing hypotheses regarding the contribution of a single locus was not an a priori consideration.
